# Symptom Improvement and Interrelated ESAS Domains Following Outpatient Palliative Care in Hungarian Cancer Patients

**DOI:** 10.3390/jcm15093532

**Published:** 2026-05-05

**Authors:** Nóra Frank, Csilla Busa, Eszter Sághy, Éva Pozsgai, Ágnes Csikós

**Affiliations:** 1 Department of Hospice-Palliative Care, Department of Primary Health Care, University of Pécs Medical School, Rákóczi Street 2, 7623 Pécs, Hungary; 2 Faculty of Health Sciences, Doctoral School of Health Sciences, University of Pécs, Szepesy Ignác Street 1–3, 7621 Pécs, Hungary; 3 Faculty of Pharmacy, University of Pécs, Rókus Street 2, 7624 Pécs, Hungary; 4 Department of Public Health Medicine, University of Pécs Medical School, Szigeti Street 12, 7624 Pécs, Hungary

**Keywords:** outpatient palliative care, early symptom management, interrelated symptoms, symptom assessment, ESAS, minimal clinically important difference, cancer symptoms, pain management, dyspnea, symptom clusters

## Abstract

**Background:** Outpatient palliative care effectively alleviates symptom burden in advanced cancer patients, yet data from Central–Eastern Europe remain scarce. This retrospective study examined changes in revised Edmonton Symptom Assessment Scale (ESAS) scores from initial outpatient palliative consultation to first follow-up in Hungarian cancer patients, assessing clinically meaningful improvement and inter-symptom associations. **Methods:** Revised ESAS scores from 119 patients attending an outpatient palliative care clinic (2017–2020) were analyzed using paired baseline and first follow-up assessments (7–30 days). Symptom changes (Time 2–Time 1) were evaluated using Wilcoxon signed-rank tests. Clinically meaningful improvement was assessed with minimal clinically important difference thresholds (0.5× baseline SD). Sankey diagrams visualized symptom transitions, and multivariable linear regression examined inter-symptom associations. **Results:** Baseline pain was highest (mean 6.29, median 7), followed by fatigue, sleep disorder, and impaired well-being. At follow-up, significant reductions were observed in pain (mean 4.52, *p* = 0.001), nausea, dyspnea, constipation, sleep disorder, depression, and anxiety (all *p* < 0.05). Sankey diagrams showed shifts from severe to mild/moderate pain (50% to 24%) and constipation. Clinically meaningful improvement occurred in pain, nausea, and constipation, with 59–65% achieving ≥1-point pain reduction. Regression analyses showed that pain reduction was associated with concurrent improvements in sleep disorder (β = 0.31), depression (β = 0.20), fatigue (β = 0.20), and anxiety (β = 0.14), while dyspnea reduction was associated with concurrent improvements in depression (β = 0.22) and anxiety (β = 0.14). **Conclusions:** Outpatient palliative care in Hungarian cancer patients resulted in clinically meaningful symptom reductions, particularly pain and dyspnea. Improvements in these core symptoms were associated with concurrent improvements in other symptom domains, underscoring the clinical relevance of inter-symptom associations and supporting early, integrated outpatient palliative care and symptom cluster-based management.

## 1. Introduction

Palliative care is a holistic approach intended to support patients with serious health issues, typically in the advanced or terminal stages of life, while also addressing the needs of their families and caregivers [1,2,3]. It focuses on improving quality of life through coordinated physical, psychological, social, and spiritual support delivered by multidisciplinary teams. Given that over 40 million individuals globally require palliative care—a figure expected to grow with population ageing and rising chronic illness—efficient service models are increasingly important [2]. Outpatient palliative care has become a key component of this framework, enabling timely assessment and symptom management outside the inpatient setting [1,2,3]. Early integration is associated with improved symptom control, better communication regarding goals of care, enhanced preparedness for end-of-life decisions [4], and reduced hospital admissions and healthcare costs [2,4,5]. Various outpatient models exist internationally, including integrated oncology–palliative clinics, co-located services, and standalone outpatient centers [6].

Hospice–palliative care has been present in Hungary for 30 years, with substantial expansion in the past 15 years [7,8,9]. There are 92 services: 26 inpatient facilities (416 beds, 411 adult beds, five children’s beds), 60 home care groups (57 adult, one mixed and two children’s providers), two inpatient consult services and four palliative outpatient clinics. Hungary currently ranks mid-range in Europe [10]. However, outpatient palliative care remains scarce, creating a gap in ambulatory symptom management despite the more broad availability of home hospice and inpatient services [8,9,10]. In 2012, the University of Pécs established the first hospice–palliative care department in the country [8] followed by the establishment of an outpatient palliative care center in 2014 within the Department of Oncotherapy. This integrated model—combining home hospice, outpatient clinics, inpatient palliative beds, and inpatient consult service—offers a unique structure for providing care aligned with patient needs and disease trajectory.

Early palliative and supportive care, including routine symptom screening, should ideally begin at the time of diagnosis of incurable disease and be continuously tailored to patients’ evolving needs [11]. Evidence suggests that timely identification and management of symptom burden may improve not only quality of life but also clinical outcomes, including survival in selected patient populations [12]. Symptom burden in advanced cancer is substantial, with patients frequently experiencing pain, fatigue, anorexia, cachexia, dyspnea, nausea, constipation, anxiety, depression, and sleep disturbance, all of which impair functioning [13,14,15,16,17]. In this context, systematic assessment of supportive care needs is essential, as these symptoms are often under-recognized and undertreated in routine oncology practice. Certain symptoms tend to occur together in clinically recognizable symptom clusters, which are influenced by primary tumor type, disease stage, and treatment burden, and collectively exacerbate patient distress [18]. From a clinical perspective, recognition of these interrelated symptom patterns is important because targeted interventions addressing key symptoms within a cluster (e.g., pain or dyspnea) may result in broader improvements across multiple related symptoms, thereby supporting a more integrated and efficient supportive care approach [18]. Early integration of palliative and supportive care and systematic symptom screening may help identify patients with high symptom burden and improve timely access to specialist services.

Systematic, rapid, and reliable assessment tools are therefore essential for identifying unmet needs and guiding timely interventions. The Edmonton Symptom Assessment Scale (ESAS), developed at the Edmonton General Hospital in 1991, is the most widely used and validated symptom assessment tool in palliative care [19,20]. It evaluates nine common symptoms, with an optional tenth patient-specific item, using an 11-point numerical rating scale from 0 to 10 [19,21]. Revisions such as the ESAS-r have refined item definitions and incorporated additional symptoms, including constipation and sleep disturbance [22]. ESAS is quick to administer, allows for efficient monitoring of symptom trends, and supports clinical decision-making in outpatient settings [23,24,25,26]. In Hungary, ESAS is routinely used in outpatient palliative care and represents a practical tool for integrating supportive care needs assessment into clinical workflow.

Several international studies have evaluated changes in ESAS symptom scores after outpatient palliative care consultations. Kang et al. reported the magnitude of symptom change at first follow-up after outpatient palliative visits in a cancer cohort [27]. A Brazilian study (n = 232) documented overall decreases in symptom distress, including pain [28], and Rafaqat et al. demonstrated significant post-consultation reductions in pain, depression, and anxiety in Pakistani patients (reported *p* < 0.001) [29]. However, it should be added that studies from the Republic of Korea and the United States have shown that although outpatient palliative consultations are associated with reductions in pain, fatigue, and psychological symptoms, many patients continue to exhibit moderate-to-high symptom burden at follow-up, indicating the need for ongoing management [24,25,26,27].

Literature supports ESAS as a responsive instrument for detecting short-term symptom changes after outpatient palliative interventions. To our knowledge, no studies have examined follow-up ESAS changes in the Central–Eastern European region, and few have assessed associations between changes in individual symptoms using regression analysis. Generating such region-specific data is particularly relevant in Hungary, where outpatient palliative services remain limited and symptom profiles may differ from those reported in other settings.

Therefore, our retrospective study aimed to evaluate changes in revised ESAS symptom scores between the initial outpatient palliative care consultation, and the first follow-up visit in a cohort of Hungarian cancer patients. ESAS was used as a standardized instrument to assess symptom burden and its changes over time in the context of outpatient palliative care outcomes. Our aims also included estimating the proportion of patients achieving clinically meaningful improvement and assessing associations between changes in individual symptoms.

## 2. Materials and Methods

### 2.1. Setting and Study Design

This retrospective study was conducted at the University of Pécs Clinical Center, Department of Oncotherapy in Hungary, which functions as a large regional cancer center, receiving patients from across the Transdanubian region. An outpatient palliative care clinic provides supportive symptom management for cancer patients. All relevant patient data, including baseline patient characteristics and results of ESAS surveys, were retrieved from the e-Medsolution hospital database.

Altogether, 800 ESAS surveys from 331 cancer patients attending the outpatient palliative care clinic between January 2017 and December 2020 were initially collected. Patients with only one completed survey or with >30 days between their first and second surveys were excluded, resulting in a final sample of 119 patients, each with two ESAS assessments conducted 7–30 days apart. The 7–30-day interval was selected to capture clinically meaningful short-term changes following the initial consultation while maintaining temporal proximity between assessments, consistent with prior outpatient palliative care studies [29,30]. Exclusion criteria therefore included: (1) only one available ESAS survey, and (2) >30-day interval between Time 1 and Time 2 assessments.

Ethical approval prior to this study was obtained from the University of Pécs Regional Research Ethics Committee (approval number: 9784-PTE2024).

### 2.2. Variables

The revised ESAS items were used as described by Hannon et al. [22], which includes items of pain, nausea, dyspnea, constipation, appetite, sleep disorder, fatigue, depression, anxiety, and well-being scored from 0 to 10, with higher scores indicating more severe symptoms. Each ESAS score at Time 1 and Time 2 was treated as a separate continuous variable for analysis. An additional variable was created for each item, capturing the score changes in time, computed by subtracting the Time 1 score from Time 2. These variables range between −10 and 10, with negative values indicating symptom improvement. The summaries of the item change variables include data only for patients with a non-zero baseline value. The number of days between the two surveys was also included as a variable in the regression analysis, alongside data collected on patient demographics (age and sex) and main diagnosis (primary cancer site).

### 2.3. Statistical Analysis

Statistical analyses were performed using RStudio (version 2022.02.1).

Symptoms were categorized based on the ESAS scores: mild symptoms were represented by ESAS scores of 0 to 3, moderate symptoms by scores of 4 to 6, and severe symptoms by scores of 7 to 10. The significance of differences between Time 1 and Time 2 ESAS item means was assessed using the Wilcoxon signed-rank test, due to non-normal distributions. A difference was deemed significant at a *p*-value of ≤0.05. Missing ESAS item values were handled using complete case analysis; each comparison was performed on all patients with valid paired scores for that item, without imputation.

The transfer of patients between three ESAS symptom categories (mild–moderate–severe) of pain and constipation over time was visualized using Sankey diagrams. These diagrams emphasized the major direction of transfers during the study [31].

The minimal clinically important difference (MCID) was estimated using the distribution-based approach of 0.5× baseline standard deviation, as described by Hui et al. and Sedaghat et al. [32,33]. This method has been validated specifically for ESAS items in cancer populations, where anchor-based and distribution-based estimates have been shown to converge, supporting its use as a clinically grounded threshold. Proportions of patients with improved scores and the mean change were calculated for patients with non-zero baseline scores and compared to the MCID value. Changes with a mean exceeding the MCID were considered clinically significant.

Multiple linear regression models were then constructed to examine associations between changes in these primary symptoms and concurrent changes in sleep disorder, fatigue, depression, and anxiety. Age and sex were included as covariates to adjust for potential confounding effects. To account for variability in the interval between assessments, the number of days between Time 1 and Time 2 was included as a covariate in all regression models.

To explore whether the associations between changes in pain and concurrent changes in other symptom domains varied by baseline pain severity, a pre-specified sensitivity analysis was conducted by re-running the four regression models separately in two strata defined by baseline pain score: mild (ESAS 0–3) and moderate to severe (ESAS 4–10), consistent with the standard ESAS severity categories applied elsewhere in this study.

## 3. Results

### 3.1. ESAS Score Changes in Outpatient Palliative Care

Our sample comprised 119 cancer patients (55 male and 64 female) with a median age of 65 years. The most common primary cancer sites were gastrointestinal and lung cancers (Table 1).

Table 2 summarizes ESAS scores at the initial outpatient palliative consultation (Time 1) and at follow-up within 7–30 days (Time 2).

At baseline, pain had the highest symptom burden, with a median score of 7 and a mean of 6.29. Fatigue (median 5), well-being (median 4), and sleep disorder (median 4) were also elevated. At follow-up, pain decreased to a median of 4 and a mean of 4.52 (*p* < 0.001). Statistically significant improvements were also observed for nausea (*p* = 0.036), dyspnea (*p* = 0.023), constipation (*p* = 0.004), sleep disorder (*p* < 0.001), depression (*p* = 0.017), and anxiety (*p* = 0.042). Changes in appetite (*p* = 0.784), fatigue (*p* = 0.125), and well-being (*p* = 0.072) were not statistically significant.

**Figure 1:**
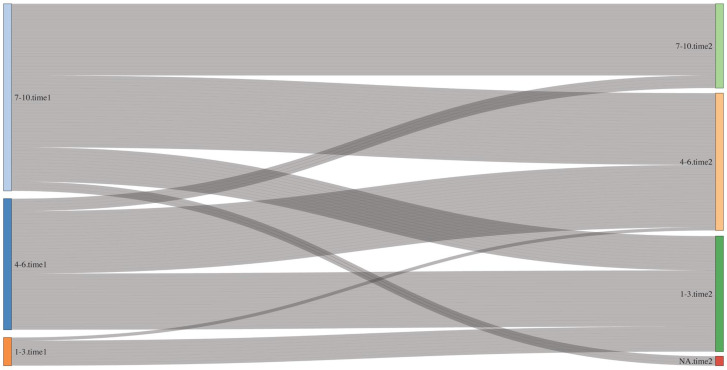
Sankey diagram showing changes in pain severity categories between initial and follow-up ESAS assessments (Time 1 → Time 2; patients with zero scores excluded).

Figure 1 shows changes in pain severity categories. Unlike summary statistics, which capture average score change at the group level, the Sankey diagram illustrates the volume and direction of individual patient transitions across severity thresholds: at Time 1, 60/119 patients (50.42%) reported severe pain (7–10), decreasing to 28 patients (23.52%) at Time 2, while the proportion with mild pain (0–3) increased from 17 (14.28%) to 46 (38.35%), indicating that a substantial number of patients crossed the severe-to-moderate threshold following the initial consultation.

**Figure 2:**
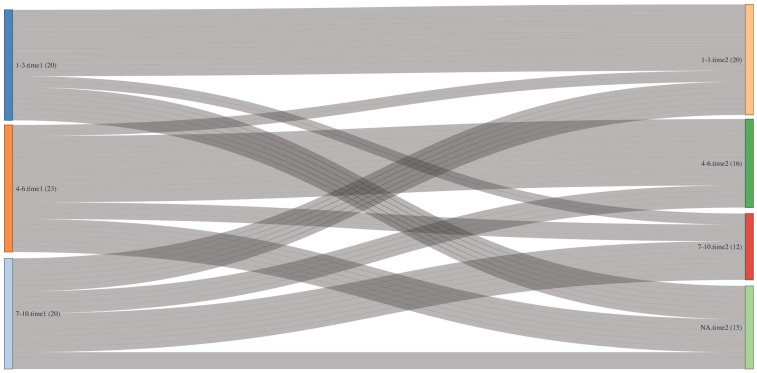
Sankey diagram showing changes in constipation severity categories between initial and follow-up ESAS assessments (Time 1 → Time 2; patients with zero scores excluded).

Constipation category changes are shown in Figure 2. Severe constipation (7–10) decreased from 20/119 patients (16.80%) to 12 (10.08%), and moderate constipation (4–6) from 23 (19.32%) to 16 (13.44%). The Sankey diagram illustrates that the improvement in constipation was characterized primarily by transitions from the severe and moderate categories toward milder scores, a pattern of individual-level change that would not be apparent from group means alone.

### 3.2. Minimal Clinically Important Difference (MCID) and Symptom Improvement

Table 3 presents the proportion of patients achieving one-point and two-point improvements, alongside mean changes and calculated MCIDs.

Clinically meaningful mean improvements (|mean change| > MCID) were observed for pain, nausea, and constipation. Pain showed the largest relative improvement, with 58.56% achieving ≥1-point reduction and 49.55% achieving ≥2-point reduction. Constipation also showed improvement (50.79% and 44.44%, respectively). Sleep disorder showed moderate improvement, though the mean change did not exceed the MCID.

### 3.3. Associations Between Symptom Changes

Multiple linear regression models were used to examine associations between changes in pain and dyspnea scores and concurrent changes in sleep disorder, depression, fatigue, and anxiety. Days between assessments, age, and sex were included as covariates.

As shown in Table 4 (n = 106–108), change in pain score was significantly associated with all four dependent variables: sleep disorder (β = 0.31, *p* < 0.001), depression (β = 0.20, *p* < 0.001), fatigue (β = 0.20, *p* = 0.004), and anxiety (β = 0.14, *p* = 0.002). Greater reductions in pain were associated with greater concurrent improvements in these symptoms.

Change in dyspnea score was significantly associated with changes in depression (β = 0.22, *p* < 0.001) and anxiety (β = 0.14, *p* = 0.020), but not with sleep disorder or fatigue. None of the covariates (age, sex, or days between assessments) had significant effects (Table 4).

In the stratified sensitivity analysis by baseline pain severity (Supplementary Table S1), the pattern of associations observed in the full sample was largely driven by patients with moderate-to-severe baseline pain (ESAS 4–10, n = 89–91). In this stratum, change in pain score was significantly associated with concurrent changes in sleep disorder (β = 0.28, *p* = 0.002), depression (β = 0.19, *p* = 0.001), and fatigue (β = 0.25, *p* = 0.003), while change in dyspnea score was significantly associated with concurrent changes in depression (β = 0.23, *p* = 0.001) and anxiety (β = 0.17, *p* = 0.008), consistent with the full-sample findings. In the mild baseline pain stratum (ESAS 0–3, n = 17), point estimates for the association between pain change and depression (β = 0.30, *p* = 0.003) and anxiety (β = 0.20, *p* < 0.001) were statistically significant and of comparable magnitude to the moderate/severe stratum; however, given the very small sample size in this group, these estimates should be interpreted with caution. Wide confidence intervals and limited statistical power preclude firm conclusions regarding stratum-specific differences, and these findings should be confirmed in larger prospective studies.

## 4. Discussion

In this study of Hungarian outpatient palliative care cancer patients, we found substantial reductions in ESAS-measured symptom intensity—notably pain, constipation, sleep disorder, nausea, dyspnea, depression, and anxiety—between the first consultation and first follow-up. To our knowledge, this is the first report from a Central–Eastern European setting describing ESAS score changes after outpatient palliative consultation, and the first to explore associations between changes in pain, dyspnea and concurrent improvements in other symptoms. We also applied MCID thresholds and Sankey diagrams to quantify and visualize clinically meaningful ESAS symptom changes, providing a patient-centered perspective.

At baseline, pain was the most severe symptom in our cohort (mean ESAS pain 6.29, median 7), exceeding values reported in many outpatient palliative settings. For example, in a large retrospective outpatient cohort by Kang et al. (n = 1612) the mean baseline pain was 5.4, declining to 4.6 at first follow-up (*p* < 0.0001). Studies from Brazil and Italy registered similarly lower baseline pain means (4.04 and 5.3, respectively) [28,34], while even lower values were reported according to a study conducted in Canada with 92 patients, where the mean baseline pain score was 3.43 with a median of 3, and a study conducted in Norway with 51 patients, which found a mean pain score of 3.1 with a standard deviation of 2.6 [35,36]. The exceptionally high pain scores observed at first visit may indicate delayed referral to outpatient palliative care in Hungary—a problem previously noted in national analyses highlighting under-awareness of palliative services among patients and some healthcare providers [37]. Furthermore, suboptimal use of available pain-management resources—whether due to systemic, organizational, or educational gaps—might contribute to the elevated symptom burden.

Following the initial outpatient consultation, we observed statistically significant reductions in multiple symptoms—pain, constipation, sleep disorders, nausea, dyspnea, depression, and anxiety—within a 7–30 day follow-up interval. These findings align with outcomes reported in other outpatient palliative care contexts. For instance, in a prospective study by Greer et al., ESAS scores for pain, fatigue, nausea, depression, anxiety, dyspnea, insomnia, and constipation improved significantly at 1 week and remained improved at 1 month [38]. Similarly, a retrospective study of advanced prostate cancer outpatients (n = 55) documented significant reductions in pain, fatigue, drowsiness, depression, sleep problems, well-being and anxiety after follow-up [15,39]. In a Jordanian cohort of 182 cancer patients, significant decreases in pain (5.9 to 5.1, *p* = 0.004) and sleep (4.6 to 4.1, *p* = 0.007) were reported [40]. Additionally, in a large multicenter review of 444 advanced-cancer outpatients with moderate-to-severe nausea, 61% experienced significant improvement of nausea after palliative consultation (*p* < 0.001) [41]. These converging findings—across cancer types, care settings, and patient populations—including our study, reinforce that outpatient palliative interventions are associated with meaningful reductions in symptom burden.

By applying MCID thresholds and reporting the proportion of patients achieving ≥ 1-point reductions, we documented clinically meaningful improvement [32]. In line with this, previous large outpatient palliative cohorts (e.g., Kang et al., 2013, n = 1612) reported that 52–74% of patients experienced at least a 1-point decrease in any ESAS item at first follow-up; this reflects improvement in at least one symptom rather than specific items such as pain or constipation [27]. Furthermore, in an integrative oncology outpatient cohort, patients with baseline ESAS scores ≥ 4 and follow-up within 30 days demonstrated statistically and clinically significant reductions across all symptoms, with ≥1-point improvement rates ranging from 49% to 75%, highlighting meaningful improvement among those with moderate-to-severe baseline symptoms [42].

Sankey diagrams are a method originally developed for representing flow processes and are widely used to visualize transitions between states over time, making them particularly suitable for depicting longitudinal changes in patient-reported outcomes [31]. This method, rarely used in outpatient ESAS studies, provides a clear visualization of individual symptom trajectories [31]. By showing the proportion of patients moving into lower-severity categories, these diagrams complement MCID thresholds and offer an interpretable depiction of clinically meaningful change beyond summary statistics [31]. Building on the MCID analyses, the Sankey diagrams in our study illustrated how patients shifted across ESAS severity categories, including transitions from severe-to-moderate or mild pain, and from moderate-to-mild constipation.

Constipation—a frequent side effect of opioid therapy in palliative cancer care—also merits attention. Opioid-induced constipation (OIC) affects an estimated 52–87% of cancer patients on opioids, causing considerable discomfort and reduced quality of life [43,44]. The average constipation score at the first appointment was 3.05, closely mirroring Follwell et al.’s findings, where the mean score was 3.9 [38]. Although opioid use was not systematically recorded in our dataset, the reduction in constipation scores following the initial visit may reflect improved symptom management consistent with international recommendations for early, proactive management of OIC, including bowel regimen initiation at the start of opioid therapy [43]. This indicated that proactive patient education and early initiation of evidence-based laxative therapy were effective components of opioid-related bowel management in routine outpatient practice.

In oncology patients, coexisting symptoms frequently influence one another, and a comprehensive approach addressing both somatic and psychosocial distress is often required [45]. In our regression analysis of ESAS data, reductions in pain and dyspnea were significantly associated with improvements in other symptoms, such as sleep disorder, fatigue, anxiety and depression. Specifically, change in pain was independently associated with improvements in all four symptom domains, while reduction in dyspnea was linked to decreases in anxiety and depression; Bruera et al. similarly reported a significant association between anxiety and dyspnea intensity [46]. Together with evidence that anxiety influences the perception and expression of dyspnea in cancer patients [47] and may both contribute to and result from dyspnea [46,48], these findings support a bidirectional, multidimensional interaction between the two symptoms. These findings are consistent with the concept of symptom clusters in advanced cancer, where multiple symptoms tend to co-occur and interact [18,49]. From a clinical standpoint, the observed associations between changes in pain and dyspnea and concurrent changes in other symptom domains suggest that improvements in core symptoms may accompany broader multidimensional benefits, though whether this reflects a causal relationship cannot be determined from this observational design. The statistically significant associations identified in our study nonetheless highlight the potential value of a holistic and multidimensional symptom management strategy in outpatient palliative care [49].

Such findings may have direct implications for clinical practice in Hungary and similar healthcare systems, where access to specialist palliative care may occur relatively late in the disease trajectory. Earlier integration of structured symptom screening and supportive care pathways within oncology services could facilitate earlier identification of high symptom burden and may help reduce delays in referral to specialist palliative care services.

Early palliative care personalised around patients’ needs, together with the prevention and management of cancer- and treatment-related symptoms (supportive care), are key components of improving patients’ quality of life and have been associated with clinical outcomes, including survival [50]. For patients with uncontrolled symptoms and impaired quality of life, national healthcare systems should ensure timely access to integrated supportive and palliative care interventions [51,52,53].

In alignment with ASCO guidelines [52], the development of hospice and palliative care services in Hungary is currently ongoing with international support, aiming to improve quality of care and ensure equitable access. In this context, integrated palliative care units are planned between 2024 and 2028 within participating hospitals. The professional framework of these developments is based on an integrated care model developed at the University of Pécs, which combines outpatient, inpatient, and consultative palliative services to better align care delivery with patient needs and disease trajectory [53].

### Limitations

This study has several limitations that should be considered when interpreting the findings. The retrospective, single-center design may limit the generalizability of the results to other outpatient palliative care populations and healthcare systems. In addition, the study lacked a control group, which restricts causal interpretation of observed symptom changes. The follow-up interval was relatively short (7–30 days) and influenced by clinical condition, patient needs, compliance, and accessibility of the specialist clinic, introducing variability in outcome measurement. Patients who returned outside this interval were typically those with lower palliative care needs, contributing to selection effects. This variability, together with the non-random timing of follow-up assessments, limits comparability between earlier and later follow-up and precludes determination of whether observed changes reflect treatment effects or natural symptom fluctuations.

The study population included patients with heterogeneous cancer types, which may influence symptom burden and trajectories. The sample size did not allow for robust stratified analyses by tumor type; therefore, potential differences across cancer subgroups could not be examined. As this was a retrospective real-world cohort study including all eligible patients attending the clinic during the study period, no formal a priori power analysis was performed.

Although ESAS is a validated and widely used tool for symptom assessment, it relies on patient self-report and may be subject to reporting bias. It is currently the only standardized symptom assessment scale routinely used in Hungary, limiting methodological triangulation. Requiring two completed ESAS assessments within 7–30 days resulted in a selected cohort, introducing potential selection, survivorship, and engagement bias and further limiting generalizability. Subgroup analyses of less frequent symptoms (e.g., constipation) were additionally limited by reduced statistical power.

Nine symptom comparisons were performed using Wilcoxon signed-rank tests at α = 0.05. While no correction for multiple comparisons was applied—as each test addresses a distinct clinical question rather than a joint hypothesis—the possibility of at least one false positive cannot be excluded. Findings for nausea (*p* = 0.036), dyspnea (*p* = 0.023), depression (*p* = 0.017), and anxiety (*p* = 0.042), in particular, should be interpreted with caution and confirmed in future prospective studies. Additionally, missing ESAS item values were handled using complete case analysis. Although the proportion of missing data per item was small, bias cannot be fully excluded if data were not missing completely at random.

The MCID was estimated using a distribution-based approach which assumes relative homogeneity of clinically meaningful change across baseline severity strata; item-level estimates may therefore not fully capture variability in meaningful change among patients with mild versus severe baseline symptoms. Finally, the small proportion of patients with mild baseline pain (ESAS 0–3, n = 17) limited the statistical power of stratified analyses examining whether inter-symptom associations vary by baseline pain severity, and stratum-specific estimates should therefore be interpreted with caution.

## 5. Conclusions

In this study of Hungarian cancer patients with palliative needs, we observed substantial and clinically meaningful reductions in ESAS-measured symptom intensity, particularly pain, constipation, sleep disorder, nausea, dyspnea, depression, and anxiety, following outpatient palliative care. By applying minimal clinically important difference thresholds and using Sankey diagrams, we provided a patient-centered visualization of symptom improvement, enhancing clinical interpretability beyond average scores.

To our knowledge, this is the first report from a Central–Eastern European setting documenting ESAS score changes following initial outpatient palliative consultation, and the first to explore inter-symptom associations, suggesting that improvements in core symptoms, such as pain and dyspnea, may be associated with improvements in other symptoms including sleep disorder, fatigue, anxiety, and depression.

These findings underscore the practical value of early, integrated outpatient palliative interventions in reducing symptom burden and improving multidimensional quality of life. They also highlight pain and dyspnea as priority targets within symptom clusters, supporting the potential value of considering symptom clusters in palliative care planning. Finally, the study implies the need for continued education of healthcare professionals regarding pain management and increased awareness relating to inter-symptom associations of outpatient palliative services in regions where such data have been lacking.

## Figures and Tables

**Table 1:** Demographic and clinical characteristics of outpatient palliative care patients (n = 119).

*Patient Characteristics*	*n* (%)
***Age (years)***	
*Mean (SD)*	65.46 (11.49)
*Median (min–max)*	65 (20–90)
***Sex***	
*Male*	55 (46.22%)
*Female*	64 (53.78%)
***Primary cancer site***	
*Breast*	10 (8.40%)
*Gastrointestinal*	44 (36.97%)
*Genitourinary*	8 (6.72%)
*Head and neck*	14 (11.76%)
*Gynaecological*	12 (10.08%)
*Lung*	22 (18.49%)
*Other*	7 (5.88%)
*Missing data*	2 (1.68%)

**Table 2:** Comparison of ESAS symptom scores at initial consultation (Time 1) (n = 119) and follow-up (Time 2) (n = 119).

	*Time 1*			*Time 2*			*Significance Between Means*	
*ESAS Items*	n	Mean (sd)	Median (Min–Max)	n	Mean (sd)	Median (Min–Max)	*p* Value	
*Pain*	119	6.29 (2.76)	7 (0–10)	118	4.52 (2.68)	4 (0–10)	<0.001	***
*Nausea*	113	1.59 (2.31)	0 (0–10)	117	1.26 (2.24)	0 (0–10)	0.036	*
*Dyspnea*	112	2.23 (2.85)	0 (0–10)	115	1.86 (2.64)	0 (0–10)	0.023	*
*Constipation*	111	3.05 (3.31)	2 (0–10)	115	2.19 (2.78)	1 (0–10)	0.004	**
*Appetite*	112	3.74 (3.06)	3 (0–10)	117	3.72 (3.02)	3 (0–10)	0.784	
*Sleep disorder*	112	3.91 (3.09)	4 (0–10)	115	3.04 (2.91)	3 (0–10)	<0.001	***
*Fatigue*	112	5.17 (2.76)	5 (0–10)	117	4.86 (2.78)	5 (0–10)	0.125	
*Depression*	112	1.97 (2.45)	1 (0–10)	118	1.56 (2.25)	0 (0–10)	0.017	*
*Anxiety*	112	2.4 (2.5)	2 (0–10)	118	2.12 (2.4)	1.5 (0–10)	0.042	*
*Well-being*	109	4.26 (2.62)	4 (0–10)	112	3.99 (2.4)	4 (0–10)	0.072	

n: number of patients. sd: standard deviation. Notes: * *p* < 0.05; ** *p* < 0.01; *** *p* < 0.001.

**Table 3:** Minimal clinically important differences (MCIDs) and proportions of patients achieving ≥1- and ≥2-point improvements for each ESAS item.

*ESAS Item*	*n*	*Change* ≤ −1 (%)	*Change* ≤ −2 (%)	*Mean Change*	*MCID*
*Pain*	**111**	**65** (**58.56%**)	**55** (**49.55%**)	**−1.95**	**−1.38**
*Nausea*	**48**	**25** (**52.08%**)	**19** (**39.58%**)	**−1.29**	**−1.16**
*Dyspnea*	53	27 (50.94%)	17 (32.08%)	−1.11	−1.43
*Constipation*	**63**	**32** (**50.79%**)	**28** (**44.44%**)	**−1.87**	**−1.65**
*Appetite*	86	22 (25.58%)	15 (17.44%)	−0.22	−1.53
*Sleep disorder*	82	45 (54.88%)	35 (42.68%)	−1.37	−1.54
*Fatigue*	104	27 (25.96%)	18 (17.31%)	−0.47	−1.38
*Depression*	61	20 (32.79%)	12 (19.67%)	−0.75	−1.23
*Anxiety*	71	22 (30.99%)	14 (19.72%)	−0.52	−1.25
*Well-being*	100	31 (31%)	13 (13%)	−0.49	−1.31

n: number of patients. Notes: Clinically important item changes are in bold, measured by a greater value of absolute mean change over MCID.

**Table 4:** Multiple linear regression analysis of associations between concurrent changes in pain and dyspnea and concurrent changes in sleep disorder, depression, fatigue, and anxiety (n = 119).

	Change in Sleep Disorder Score		Change in Depression Score		Change in Fatigue Score		Change in Anxiety Score	
	*β*	*p*	*β*	*p*	*β*	*p*	*β*	*p*
(Intercept)	0.01	0.989	−0.66	0.345	1.76	0.116	−0.50	0.488
Change in Pain Score	**0.31**	**<0.001**	**0.20**	**<0.001**	**0.20**	**0.004**	**0.14**	**0.002**
Change in Dyspnea Score	0.12	0.186	**0.22**	**<0.001**	0.15	0.114	**0.14**	**0.020**
Days Between Assessments	−0.01	0.663	0.01	0.338	0.03	0.257	0.02	0.267
Age (years)	0.00	0.983	0.01	0.495	−0.03	0.053	0.01	0.498
Sex (female)	−0.15	0.684	0.04	0.856	−0.06	0.866	−0.41	0.096
Observations	106		108		108		108	

Bold values indicate statistical significance at *p* < 0.05. *R^2^/R^2^*-adjusted values by outcome: sleep disorder 0.196/0.156; depression 0.319/0.286; fatigue 0.169/0.128; anxiety 0.205/0.166. *β*: standardized regression coefficient.

## Data Availability

The datasets used and/or analyzed during the current study are available from the corresponding author on reasonable request.

## Supplementary Materials

The following supporting information can be downloaded at: https://www.mdpi.com/article/10.3390/jcm15093532/s1, Supplementary Table S1: Stratified multivariable linear regression: associations between changes in pain and dyspnea and concurrent changes in sleep disorder, depression, fatigue, and anxiety, by baseline pain severity.

## Abbreviations

ESAS	Edmonton Symptom Assessment Scale
OIC	Opioid-Induced Constipation
MCID	Minimal Clinically Important Difference
