# Supplementary material for: Symptom Improvement and Interrelated ESAS Domains Following Outpatient Palliative Care in Hungarian Cancer Patients
Source: J Clin Med. 2026 May 5;15(9):3532. doi: 10.3390/jcm15093532 (PMC13165243; doi:10.3390/jcm15093532)
Supplement: Supplementary file 1 [file jcm-15-03532-s001.zip › jcm-4236682-supplementary.pdf]

Supplementary Table S1. Stratified multivariable linear regression: associations between changes in pain and dyspnea and concurrent changes in sleep disorder, depression, fatigue, and anxiety, by baseline pain severity.

Mild baseline pain (ESAS 0–3, n = 17)

	**Change in Sleep Disorder Score**			**Change in Depression Score**			**Change in Fatigue Score**			**Change in Anxiety Score**		
	β	***95% CI***	***p***	***β***	***95% CI***	***p***	***β***	***95% CI***	***p***	***β***	***95% CI***	***p***
(Intercept)	2.32	-2.09 – 6.74	0.271	-0.22	-0.98 – 0.54	0.537	-1.12	-4.40 – 2.16	0.468	0.21	-0.16 – 0.58	0.235
Change in Pain Score	0.71	-0.29 – 1.71	0.148	**0.30**	**0.12 – 0.47**	**0.003**	-0.03	-0.78 – 0.71	0.929	**0.20**	**0.11 – 0.28**	**<0.001**
Change in Dyspnea Score	0.33	-3.49 – 4.15	0.852	0.12	-0.54 – 0.77	0.696	-0.36	-3.20 – 2.47	0.783	**-1.16**	**-1.49 – -0.84**	**<0.001**
Days Between Assessments	0.01	-0.14 – 0.16	0.872	0.00	-0.02 – 0.03	0.919	0.01	-0.10 – 0.12	0.839	-0.00	-0.01 – 0.01	0.846
Age (years)	-0.04	-0.10 – 0.02	0.152	0.00	-0.01 – 0.01	0.757	0.01	-0.04 – 0.05	0.777	-0.00	-0.01 – 0.00	0.255
Sex (female)	-0.12	-2.25 – 2.02	0.906	0.17	-0.19 – 0.54	0.325	0.49	-1.10 – 2.08	0.510	-0.01	-0.19 – 0.17	0.920
R^2 / R^2 adjusted	0.629 / 0.460			0.888 / 0.838			0.131 / -0.263			0.991 / 0.988		

Moderate/severe baseline pain (ESAS 4–10, n = 89–91)

	**Change in Sleep Disorder Score**			**Change in Depression Score**			**Change in Fatigue Score**			**Change in Anxiety Score**		
	β	***95% CI***	***p***	***β***	***95% CI***	***p***	***β***	***95% CI***	***p***	***β***	***95% CI***	***p***
(Intercept)	-0.62	-3.14 – 1.91	0.629	-0.77	-2.41 – 0.86	0.350	2.44	-0.10 – 4.98	0.060	-0.75	-2.37 – 0.87	0.358
Change in Pain Score	**0.28**	**0.10 – 0.45**	**0.002**	**0.19**	**0.08 – 0.30**	**0.001**	**0.25**	**0.09 – 0.42**	**0.003**	0.09	-0.02 – 0.19	0.103
Change in Dyspnea Score	0.15	-0.05 – 0.35	0.138	**0.23**	**0.10 – 0.35**	**0.001**	0.13	-0.06 – 0.33	0.184	**0.17**	**0.05 – 0.30**	**0.008**
Days Between Assessments	-0.02	-0.07 – 0.04	0.586	0.02	-0.02 – 0.05	0.341	0.03	-0.03 – 0.08	0.357	0.03	-0.01 – 0.06	0.149
Age (years)	0.01	-0.03 – 0.05	0.604	0.01	-0.02 – 0.03	0.526	-0.04	-0.07 – -0.00	0.050	0.01	-0.02 – 0.03	0.553
Sex (female)	-0.15	-1.00 – 0.70	0.728	0.04	-0.50 – 0.59	0.877	-0.22	-1.07 – 0.64	0.614	-0.43	-0.98 – 0.11	0.116
R^2 / R^2 adjusted	0.163 / 0.113			0.299 / 0.258			0.201 / 0.154			0.184 / 0.136		

Bold values indicate statistical significance at $p < 0.05$. β: unstandardized regression coefficient. CI: confidence interval. The mild baseline pain stratum (ESAS 0–3, n = 17) should be interpreted with caution due to limited statistical power. R^2/R^2 adjusted values reflect model fit within each stratum.
